# A comparison of methods for the measurement of adherence to antihypertensive multidrug therapy and the clinical consequences: a retrospective cohort study using the Korean nationwide claims database

**DOI:** 10.4178/epih.e2023050

**Published:** 2023-05-01

**Authors:** Minji Jung, Eunjung Choo, Sukhyang Lee

**Affiliations:** 1 Department of Urology, School of Medicine, Stanford University, Palo Alto, CA, USA; 2 Division of Clinical Pharmacy, Ajou University College of Pharmacy, Suwon, Korea

**Keywords:** Antihypertensive agent, Therapeutic adherence, Combination drug therapy

## Abstract

**OBJECTIVES:**

In observational studies, the methods used to measure medication adherence may affect assessments of the clinical outcomes of drug therapy. This study estimated medication adherence to multidrug therapy in patients with hypertension using different measurement methods and compared their impacts on clinical outcomes.

**METHODS:**

This was a retrospective cohort study using the Korean National Health Insurance Service–National Sample Cohort database (2006-2015). Adults diagnosed with hypertension who initiated multidrug antihypertensive therapy in the index year 2007 were included. Adherence was defined as over 80% compliance. Adherence to multidrug antihypertensive therapy was measured in 3 ways using the proportion of days covered (PDC) with 2 approaches to the end-date of the study observations: PDC with at least one drug (PDC_with≥1_), PDC with a duration weighted mean (PDC_wm_), and the daily polypharmacy possession ratio (DPPR). The primary clinical outcome was a composite of cardiovascular and cerebrovascular disease-specific hospitalizations or all-cause mortality.

**RESULTS:**

In total, 4,226 patients who initiated multidrug therapy for hypertension were identified. The mean adherence according to the predefined measurements varied from 72.7% to 79.8%. Non-adherence was associated with an increased risk of a primary outcome. The hazard ratios (95% confidence intervals, CIs) primary outcomes varied from 1.38 (95% CI, 1.19 to 1.59) to 1.44 (95% CI, 1.25 to 1.67).

**CONCLUSIONS:**

Non-adherence to multidrug antihypertensive therapy was significantly associated with an increased risk of a primary clinical outcome. Across the varying estimates based on different methods, medication adherence levels were similar. These findings may provide evidence to support decision-making when assessing medication adherence.

## GRAPHICAL ABSTRACT

[Fig f4-epih-45-e2023050]

## INTRODUCTION

Adherence to medications is defined as the extent to which a patient takes medications according to the prescribed dosage, frequency, and duration [1-4]. Non-adherence is associated with an increased risk of clinical consequences and suboptimal therapeutic effects [5-7], and it is related to an estimated US$100-300 billion annually in potentially avoidable healthcare costs, accounting for 10% of total healthcare costs [8,9]. Therefore, adherence to medication is one of the key measurements used to optimize therapeutic effects, lower the risk of clinical consequences, and decrease the health care burden.

Hypertension, one of the most common chronic diseases worldwide, is particularly challenging because patients require long-term medications to maintain their blood pressure within the target range and to prevent clinical consequences such as cardio-cerebrovascular disease (CVD) [10]. In Korea, approximately 63% (9.7 million) of patients with a diagnosis of hypertension received antihypertensive medications in 2018, approximately 4 times higher than in 2002 [11]. However, half did not adhere to their medications, with a standard threshold of 80%. Furthermore, approximately 60% needed multidrug therapy, a known risk factor for medication non-adherence related to the increased complexity of the regimen [12-14]. With the growing use of antihypertensive medications, improved adherence to these medications, especially in multidrug therapy settings, can lead to improved clinical and economic healthcare outcomes [15].

To improve adherence to multidrug therapy, it is necessary to standardize the methods used to measure adherence. However, there are 2 barriers to measuring adherence. First, numerous studies have used different adherence measurements. Currently, the proportion of days covered (PDC) is suggested by the Pharmacy Quality Alliance as the preferred measurement for adherence to antihypertensive medications [16]. The validity of the PDC in monotherapy has been proved in various studies; however, it remains unclear whether it is applicable in multidrug therapy settings [17]. Over the past decade, several concepts and definitions of adherence have been suggested. Various PDC-based methods have been used for multidrug therapy, such as PDC with at least 1 drug (PDC_with≥1_) and PDC with a duration-weighted mean (PDC_wm_) [17-19]. Recently, the daily polypharmacy possession ratio (DPPR) was proposed to assess adherence to multidrug therapy [17,20]. However, these methods have not been comprehensively compared. Second, the results of studies analyzing various approaches to medication use have been inconsistent [17,21,22]. Observational studies have reported that patients took their medications as prescribed, that patients refilled prescriptions before their previous supply was depleted, or that patients took extra doses accumulated during the observation period [21,22]. Nevertheless, the comparative validity of these results remains unclear.

In a previous study we compared the 3 abovementioned measurements of adherence to antihypertensive medications and developed an algorithm of 8 different approaches incorporating 25 alternative assumptions [22]. We suggested that PDC_with≥1_ could be the preferred option. We also found that the end-date of the study’s observation period, as with prescription-based methodologies (PxMs) or fixed period-based methodologies (FxMs), may influence the measurements of adherence. However, these findings relied on samples that used both single-drug and multidrug regimens, and we did not study them separately. Therefore, the present study aimed to compare 3 measurements of adherence, including PDC_with≥1_, PDC_wm_, and DPPR, across 2 approaches to defining the end-date of the study observations (PxM and FxM) in patients receiving multidrug antihypertensive therapy and investigate their clinical consequences.

## MATERIALS AND METHODS

### Data source

The Korean National Health Insurance Service (NHIS) provides coverage for over 97% of the Korean population [23]. The Korean National Health Insurance Service–National Sample Cohort (NHIS–NSC) database from 2006 to 2015 includes data from approximately 1 million Koreans sampled among all beneficiaries [23,24]. The NHIS-NSC contains data on patient demographics including age and sex, diagnostic records, and prescription records from both inpatient and outpatient settings. The diagnostic records were coded according to the International Classification of Diseases, 10th revision (ICD‐10). The prescription records were coded according to the Korean Drug and Anatomical Therapeutic Chemical Codes. The need for informed consent was waived, as all data in the NHIS-NSC were anonymized before the database was provided.

### Study population

**Figure 1.**
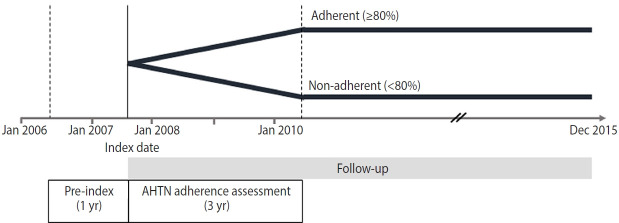
Schematic diagram of the study design. The index date was the date of the first prescription for antihypertensive medications, and adherence to the medications was assessed for 3 years from the index date. We applied inclusion/exclusion criteria to finalize the study participants using this study design. Each patient was followed from the index date until the occurrence of a study outcome, death, or the end-date of the study (December 31, 2015), whichever came first. AHTN, antihypertensive agents.

**Figure 2.**
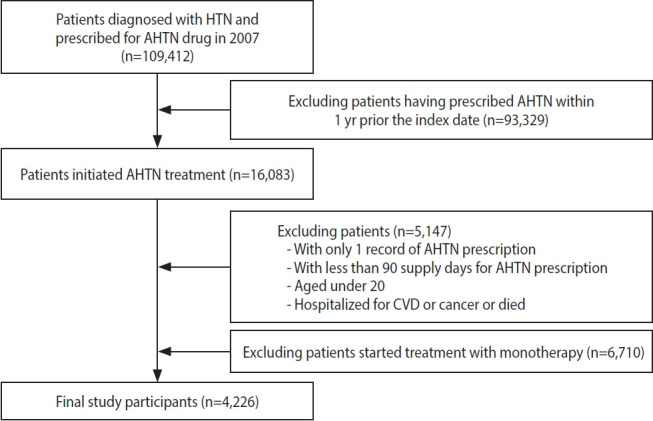
Patient selection flowchart. We excluded the following patients: (1) those who had a prescription record of antihypertensive medications within 1 year before the index date, (2) those who had a prescription record of monotherapy for antihypertensive medications, (3) those who used antihypertensive medications <90 days, (4) those who had a record of hospitalization for CVD or cancer within 1 year before the index date, and (5) those who died or developed CVD or cancer within 3 years after the index date. HTN, hypertension; AHTN, antihypertensive agents; CVD, cardio-cerebrovascular disease.

In this nationwide retrospective cohort study, we included patients aged > 20 years who had a diagnosis of hypertension and at least 2 prescriptions for antihypertensive medications (i.e., multidrug therapy) from 2 or more classes of drugs in the index year 2007. We included 5 classes of antihypertensive medications: angiotensin-converting enzyme inhibitors, angiotensin receptor blockers, beta-blockers, calcium channel blockers, and diuretics. The index date was the date of the patient’s first prescription for antihypertensive medications. We assessed adherence to the medications for 3 years from the index date (Figure 1). The following patients were excluded: (1) those who had a prescription record of antihypertensive medications within 1 year before the index date, (2) those who had a prescription record of a single antihypertensive medication (monotherapy), (3) those who had used antihypertensive medications for < 90 days, (4) those who had been hospitalized with a diagnosis of CVD or cancer within 1 year before the index date, and (5) those who died or developed CVD or cancer within 3 years after the index date (Figure 2).

### Measurements of adherence

The aim of this study was to measure the patients’ adherence to multidrug antihypertensive therapy. Multidrug antihypertensive therapy was defined as 2 or more medications from the 5 drug classes, prescribed from the index date for a period of 3 years to reflect chronic treatment. We categorized study participants into 2 groups with a cut-off value of 80%: adherent patients (≥ 80%) and non-adherent patients (< 80%) [25].

To compare the measurements of adherence to multidrug antihypertensive therapy and their clinical consequences, we included PDC_with≥1_, PDC_wm_, and DPPR, which had been analyzed in our previous study [17-20,22]. First, PDC_with≥1_ was calculated as the number of days covered by ≥ 1 prescription during the observation period, divided by the number of days from the first prescription to the end of the observation period [17,19]. Second, PDC_wm_ was calculated as the sum of values obtained by multiplying the PDC of each drug class by its duration weight, divided by the sum of the weights [17,18]. Third, DPPR was calculated as the sum of each day’s score, which was a ratio of the number of drugs available for the day to the number of drug classes to be taken for the day, divided by the number of days in the observation period [17,20]. Detailed explanations are presented in Supplementary Material 1.

In addition, we compared 2 approaches (PxM and FxM) to defining the end-date of the study, which had been shown to be a potentially significant factor when measuring adherence in previous studies [22,26-28]. The PxM approach assumes that a patient takes the medication from the first prescription fill to the end of the supply of the last refill, while the FxM approach assumes that a patient takes the medication during the entire observation period (Supplementary Material 1). We derived 6 estimates by combining the 3 adherence measurements with the 2 approaches to the end-date. We also examined the adherence estimates stratified by sex, a factor for which previous studies have reported inconsistent findings.

### Study outcome

The primary outcome was a composite of CVD-related hospitalizations and all-cause mortality. CVD was defined as a code for ischemic heart disease (ICD-10 codes I20-I25), cardiovascular diseases (ICD-10 codes I05-I09, I26-I28, I30-I52), stroke (ICD-10 codes I60-I64), and cerebrovascular diseases (ICD-10 codes I65-I69, G45-G46) [29]. The secondary outcomes were all-cause mortality, CVD-related mortality, and CVD-related hospitalization. The cause of death was obtained using the ICD-10 codes applied by Statistics Korea in this study database, and by individually linking the information using a unique personal identification number [30]. Previous validation studies have shown high positive predictive values (> 90%) for the outcomes [29,31,32]. Each patient was followed from the index date until the occurrence of a primary or secondary outcome or the end-date of the study’s observation period (December 31, 2015), whichever came first.

### Covariates

The baseline characteristics included sex, age, disability, type of health insurance, socioeconomic status, type of medical institution, type of multidrug therapy, Charlson comorbidity index (CCI) score, and history of diabetes mellitus or dyslipidemia within 1 year prior to the index date [29]. Categories were defined for age (20-49, 50-69, and > 70 years), type of health insurance (National Health Insurance or Medical Aid), socioeconomic status (high, moderate, low, and missing data), and medical institution (tertiary, secondary, clinic, and public health center). The type of multidrug therapy, based on the number of antihypertensive medication classes, was categorized as dual therapy or ≥ triple therapy. To estimate the individual medical burdens of disease, the CCI was categorized as 0, 1, or ≥ 2.

### Statistical analysis

In the main analysis, we presented mean (standard deviation) and median (interquartile range, IQR) values for adherence levels and the numbers of adherent patients. We used Cox proportional hazards analysis to model the time to the primary outcome as a dependent variable, and the measurement of adherence as the independent variable. We adjusted the model for sex and age, and fully adjusted it for sex, age, disability, type of health insurance, socioeconomic status, type of medical institution, type of multidrug therapy, CCI score, and history of diabetes mellitus or dyslipidemia. To test the goodness-of-fit for the risk models in survival analyses, we compared the Akaike information criterion (AIC) and concordance indexes including Harrell’s and Uno’s C-index. The AIC was used to evaluate predictive performance. While Harrell’s C-index has been shown to have an upward bias if a large volume of patients is censored, Uno’s C-index has shown less bias in such situations. These C-indexes were used to assess differences between the unadjusted base model and the models adjusted for the 6 measurements of adherence. A C-index of > 0.7 was considered a good model and higher estimates when compared to the base model were considered better. A p-value of < 0.05 was considered to indicate statistical significance. All data analyses were conducted using SAS version 9.4 (SAS Institute Inc., Cary, NC, USA).

### Ethics statement

This study protocol was approved by the Institutional Review Board (IRB) of Ajou University, Korea (201912-HB-EX-001). Informed consent was waived by the IRB.

## RESULTS

Among the study population of 4,226 patients who had a diagnosis of hypertension and initiated multidrug antihypertensive therapy in 2007, 47.4% were female, 37.2% were > 60 years old, 7.4% had a disability, 94.4% had National Health Insurance, and 37.9% had a high-income level. A total of 3,273 patients (77.4%) took 2 antihypertensive medications and 22.6% took ≥ 3 medications. Approximately 15.4% and 31.0% had a history of diabetes mellitus and dyslipidemia, respectively (Table 1). A comparison of the baseline characteristics between the adherent and non-adherent groups according to PDC_with≥1_, PDC_wm_, and DPPR are shown in Supplementary Materials 2-7.

**Figure 3.**
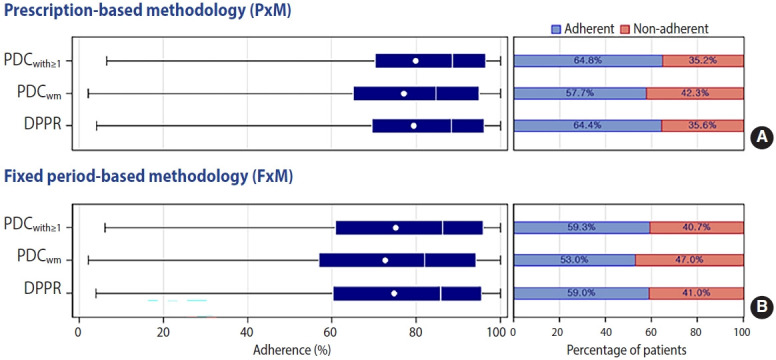
Estimates and percentage of adherence to multidrug therapy according to 6 predefined measurements. The PDC_with≥1_, PDC_wm_, and DPPR measurements using the PxM (A) and FxM (B) approaches to defining the study’s end-date showed that a total of 2,739 (64.8%) and 2,506 (59.3%), 2,440 (57.7%) and 2,240 (53.0%), and 2,720 (64.4%) and 2,493 (59.0%) patients were adherent, respectively. The mean adherence across the 6 measurements varied from 72.7% to 79.8%. PDC_with≥1_, proportion of days covered with at least one drug; PDC_wm_, duration weighted mean PDC; DPPR, daily polypharmacy possession ratio.

The results of PDC_with≥1_, PDC_wm_, and DPPR showed that, according to PxM and FxM, a total of 2,739 (64.8%) and 2,506 (59.3%), 2,440 (57.7%) and 2,240 (53.0%), and 2,720 (64.4%) and 2,493 (59.0%) patients were adherent, respectively. The mean values of adherence varied from 72.7% to 79.8% (Table 2 and Figure 3). Estimates of adherence were similar between sexes (Supplementary Material 8).

Compared to the adherent group, the non-adherent group was significantly associated with an increased risk of a primary outcome. The 6 estimates of adherence levels varied from the fully adjusted hazard ratio (HR) of 1.38 (95% confidence intervals [CI], 1.19 to 1.59) to an HR of 1.44 (95% CI, 1.25 to 1.67). The estimates of adherence related to the risks of all-cause mortality were from (HR, 1.48; 95% CI, 1.16 to 1.89) to (HR, 1.65; 95% CI, 1.30 to 2.10). The risks of CVD-related mortality varied from (HR, 1.95; 95% CI, 1.20 to 3.18) to (HR, 2.53; 95% CI, 1.52 to 4.23). The risks of CVD-related hospitalization differed from (HR, 1.40; 95% CI, 1.20 to 1.64) to (HR, 1.45; 95% CI, 1.24 to 1.70) (Table 3). In the fully adjusted models, the AIC was lower, and Harrell’s and Uno’s C-indexes were higher than 0.7 across the 6 estimates, including the 3 measurements of adherence with FxM and PxM (Table 4, Supplementary Material 9).

## DISCUSSION

This population-based cohort study compared the 3 measurements of adherence to multidrug antihypertensive therapy (PDC_with≥1_, PDC_wm_, and DPPR) across 2 approaches to defining the end-date of observations (PxM and FxM), and their comparative clinical outcomes. This study found that (1) the adherence estimates of PDC_with≥1_, PDC_wm_, and DPPR were similar, (2) these trends were consistently shown in both the PxM and FxM approaches, and (3) non-adherence was associated with a 40% increased risk of a primary outcome. To the best of our knowledge, this is the first study to compare the different approaches for measuring adherence to multidrug antihypertensive therapy over a 3-year period and to identify their clinical outcomes using a Korean nationwide claims database.

In this study, median adherence levels of > 80% to multidrug antihypertensive therapy were shown across the 6 estimates. The percentage of adherent study subjects varied from 53.0% to 64.8%. Although the Korean Society of Hypertension reported in their 2018 fact sheet that 49.5% of adults were adherent to antihypertensive medications in 2007, the index year of the current study, they did not present how adherence was measured [11]. Although the percentage has increased from 24.0% in 2002 to 72.1% in 2018 because of the growing number of patients with hypertension and a longer life expectancy, optimizing adherence to medications is paramount.

PDC_with≥1_, PDC_wm_, and DPPR showed similar adherence levels. As there were no previous studies comparing these 3 measurements in multidrug antihypertensive therapy, it is difficult to directly compare to previous studies. Our previous study of patients receiving both antihypertensive single and multidrug regimens showed similar trends [22]. However, a previous study comparing adherence to antidiabetic therapy showed different trends; the median value (IQR) of adherence was 95.9% (IQR, 72.3-100) and 77.5% (IQR, 51.8-96.4) when using PDC_with≥1_ and DPPR, respectively [33]. Although PxM showed slightly higher estimates than FxM, similar trends were shown in both. Other studies have supported our findings [18,25,34]. In studies of chronic medication therapy, the study observation window, which is the denominator in calculating adherence, was generally shorter in the FxM approach than in the PxM approach. These findings demonstrated that different measurements and approaches to evaluating medication adherence could contribute to differing results. Identifying a reasonable range of adherence levels would help minimize the sample size necessary for clinical trials or observational research, especially when enrolling subjects with chronic and multidrug therapy, who tend to be less adherent.

As expected, non-adherence to multidrug antihypertensive therapy was significantly associated with a 40% increased risk of the primary clinical outcomes. Moreover, these associations were also largely consistent across the 6 estimates for individual outcomes. Interestingly, the HRs increased with adjustments to the covariates. This could be explained by age (> 50 years), type of multidrug therapy (≥ triple therapy), and the presence of diabetes or dyslipidemia, which were negatively related to non-adherence, and positively related to the study outcomes of mortality or hospitalization (Supplementary Materials 2-7). These findings were in line with other studies [5-7,35]. A meta-analysis showed that adherence to cardiovascular medications including antihypertensive medications, antiplatelet drugs, and statins was associated with 44%, 34%, and 39% decreased risk of all-cause mortality, CVD-related mortality, and CVD-related hospitalization or myocardial infarction, respectively [35]. In a subgroup analysis, including multidrug therapy with these cardiovascular medications, non-adherence was associated with a 43% higher risk of all-cause mortality.

Although no gold standard for measuring adherence to multidrug therapy is currently available, DPPR is the most recent and theoretically the optimal measurement of adherence to multidrug therapy [36]. This method accounts for the specificity of multidrug therapy, including the number of concurrent medications used and the frequency of switching medications. The measurement of DPPR is based on daily supply information by determining how many medications are available and weighting a score by the number of medications to be taken each day. Despite its sensitive and intricate method, however, the DPPR has not been widely used. The calculations to generate estimates can be complicated, both manually and when aided by a computer, especially in long-term follow-up. This study found that the PDC_with≥1_ showed similar results to the DPPR. Thus, the more traditional and simplest method, the PDC_with≥1_, might be an alternative option to the more current and complex method, the DPPR, when measuring adherence to antihypertensive multidrug therapy.

This study had several strengths. First, we demonstrated standardized ways of comparing and validating different measurements and approaches when assessing adherence to multidrug antihypertensive therapy in patients with hypertension. Second, this study affirmed the robustness of a higher risk for primary clinical outcomes by comparing non-adherence to adherence across the different adherence measurements.

Our study also had limitations. First, the use of a claims database, which collects data for reimbursement and not for research purposes, limited our ability to validate whether patients actually took their medications as prescribed. However, our findings may help identify a reasonable range of medication adherence and the related clinical outcome risks according to how the adherence is measured. Second, as this study focused on measuring adherence to individual classes of antihypertensive medications, we were unable to compare fixed-dose combination pills to single-medication pills. Because adherence to medication is a complex phenomenon that requires comprehensive assessment, the current study needs to be expanded further. Third, due to the unique characteristics of the Korean NHIS, which is a unified nationwide single payer, we were able to conduct uninterrupted follow-up of the participants and measure their adherence to the chronic use of multidrug antihypertensive therapy. In addition, the Korean NHIS provides a relatively stable health system, which might influence medication adherence. Further studies in different health systems are necessary to validate our findings. Lastly, adherence might vary according to different types of medications and their regimens. Additional research is warranted to study adherence to other medications that are commonly used in chronic and multidrug therapy such as antidiabetic drugs.

In conclusion, the associations between non-adherence to multidrug antihypertensive therapy and the higher risk of primary clinical outcomes were largely consistent when applying the PDC_with≥1_, PDC_wm_, and DPPR measurements across 2 approaches to defining the study’s end-date (FxM and PxM). This study proposes the potential utilization of PDC_with≥1_ as a broad, practical, and reliable method, instead of other more complicated measurement options, in patients with hypertension.

## Figures and Tables

**Figure f4-epih-45-e2023050:**
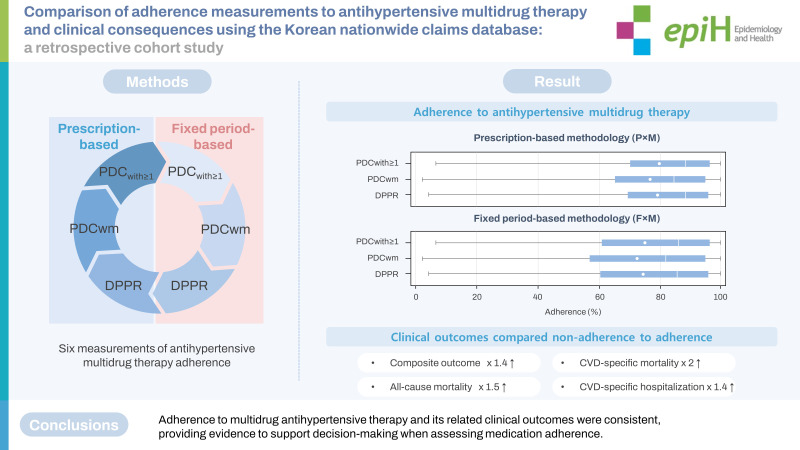


**Table 1.** Baseline characteristics of study population

Characteristics		n (%)
Overall		4,226 (100)
Sex	Male	2,224 (52.6)
	Female	2,002 (47.4)
Age (yr)	mean±SD	55.72 ±12.49
	20-39	384 (9.1)
	40-49	1,050 (24.8)
	50-59	1,220 (28.9)
	60-69	928 (22.0)
	≥70	644 (15.2)
Disability		314 (7.4)
Type of health insurance	National Health Insurance	3,988 (94.4)
	Medical Aid	238 (5.6)
Socioeconomic status	High	1,601 (37.9)
	Middle	1,431 (33.9)
	Low	919 (21.7)
	Missing data	275 (6.5)
Medical institution type	Tertiary	186 (4.4)
	Secondary	432 (10.2)
	Clinic	3,222 (76.2)
	Public health center	386 (9.1)
No. of AHTN classes	2	3,273 (77.4)
	≥3	953 (22.6)
Charlson comorbidity index	0	3,071 (72.7)
	1	800 (18.9)
	≥2	355 (8.4)
Diabetes		652 (15.4)
Dyslipidemia		1,308 (31.0)

SD, standard deviation; AHTN, antihypertensive agents.

**Table 2.** Adherence rates, according to six methodologic measurements

Adherence measures	Adherence (%)		Adherent (≥ 80%)
	Mean±SD	Median (Q1-Q3)	n (%)
PxM			
PDC_with≥1_	79.84±21.98	88.53 (70.19-96.45)	2,739 (64.8)
PDC_wm_	77.02±22.43	84.66 (65.15-94.92)	2,440 (57.7)
DPPR	79.39±22.09	88.31 (69.43-96.05)	2,720 (64.4)
FxM			
PDC_with≥1_	75.16±26.40	86.26 (60.82-95.89)	2,506 (59.3)
PDC_wm_	72.66±26.24	82.01 (56.89-94.16)	2,240 (53.0)
DPPR	74.76±26.44	85.81 (60.18-95.50)	2,493 (59.0)

SD, standard deviation; PxM, prescription-based methodology; PDC_with≥1_, proportion of days covered with at least one drug; PDC_wm_, duration weighted mean PDC; DPPR, daily polypharmacy possession ratio; FxM, fixed period-based methodology.

**Table 3.** Study outcomes compared non-adherence to adherence according to the predefined six methodologic measurements

Variables			Adherent	Non-adherent	Unadjusted model	Age, sex adjusted model	Fully adjusted model
All-cause mortality and CVD-specific hospitalization							
	PxM						
		PDC_with≥1_	471 (17.2)	315 (21.2)	1.26 (1.10, 1.46)	1.34 (1.16, 1.55)	1.40 (1.21, 1.62)
		PDC_wm_	411 (16.8)	375 (21.0)	1.27 (1.11, 1.46)	1.33 (1.16, 1.53)	1.38 (1.19, 1.59)
		DPPR	467 (17.2)	319 (21.2)	1.27 (1.10, 1.46)	1.34 (1.16, 1.55)	1.39 (1.21,1.61)
	FxM						
		PDC_with≥1_	423 (16.9)	363 (21.1)	1.29 (1.12, 1.48)	1.38 (1.20, 1.58)	1.44 (1.25, 1.66)
		PDC_wm_	369 (16.5)	417 (21.0)	1.31 (1.14, 1.51)	1.39 (1.21, 1.60)	1.44 (1.25, 1.67)
		DPPR	422 (16.9)	364 (21.0)	1.28 (1.11, 1.47)	1.37 (1.19, 1.57)	1.42 (1.24, 1.64)
All-cause mortality							
	PxM						
		PDC_with≥1_	161 (5.9)	120 (8.1)	1.39 (1.10, 1.76)	1.42 (1.12, 1.80)	1.48 (1.16, 1.89)
		PDC_wm_	137 (5.6)	144 (8.1)	1.45 (1.15, 1.83)	1.48 (1.17, 1.87)	1.56 (1.23, 1.98)
		DPPR	159 (5.8)	122 (8.1)	1.40 (1.11, 1.78)	1.43 (1.13, 1.81)	1.49 (1.17, 1.90)
	FxM						
		PDC_with≥1_	141 (5.6)	140 (8.1)	1.47 (1.16, 1.86)	1.50 (1.19, 1.90)	1.59 (1.25, 2.01)
		PDC_wm_	121 (5.4)	160 (8.1)	1.51 (1.20, 1.92)	1.56 (1.23, 1.97)	1.65 (1.30, 2.10)
		DPPR	141 (5.7)	140 (8.1)	1.45 (1.15, 1.83)	1.48 (1.17, 1.88)	1.57 (1.23, 1.99)
CVD-specific mortality							
	PxM						
		PDC_with≥1_	35 (1.3)	33 (2.2)	1.76 (1.10, 2.84)	1.82 (1.13, 2.93)	1.99 (1.22, 3.24)
		PDC_wm_	28 (1.1)	40 (2.2)	1.98 (1.22, 3.21)	2.03 (1.26, 3.30)	2.17 (1.32, 3.55)
		DPPR	35 (1.3)	33 (2.2)	1.73 (1.07, 2.78)	1.78 (1.11, 2.87)	1.95 (1.20, 3.18)
	FxM						
		PDC_with≥1_	28 (1.1)	40 (2.3)	2.12 (1.31, 3.43)	2.20 (1.36, 3.57)	2.47 (1.50, 4.07)
		PDC_wm_	23 (1.0)	45 (2.3)	2.24 (1.36, 3.71)	2.34 (1.41, 3.86)	2.53 (1.52, 4.23)
		DPPR	28 (1.1)	40 (2.3)	2.09 (1.29, 3.39)	2.17 (1.34, 3.53)	2.44 (1.48, 4.01)
CVD-specific hospitalization							
	PxM						
		PDC_with≥1_	381 (13.9)	263 (17.7)	1.30 (1.11, 1.53)	1.38 (1.18, 1.62)	1.45 (1.24, 1.70)
		PDC_wm_	334 (13.7)	310 (17.4)	1.29 (1.11, 1.51)	1.35 (1.16, 1.58)	1.40 (1.20, 1.64)
		DPPR	379 (13.9)	265 (17.6)	1.29 (1.11, 1.51)	1.37 (1.17, 1.60)	1.43 (1.22, 1.68)
	FxM						
		PDC_with≥1_	347 (13.8)	297 (17.3)	1.28 (1.10, 1.50)	1.37 (1.18, 1.60)	1.44 (1.23, 1.69)
		PDC_wm_	304 (13.6)	340 (17.1)	1.30 (1.11, 1.51)	1.38 (1.18, 1.61)	1.43 (1.22, 1.67)
		DPPR	346 (13.9)	298 (17.2)	1.27 (1.09, 1.49)	1.36 (1.17, 1.59)	1.42 (1.22, 1.67)

Values are presented as number (%) or hazard ratio (95% confidence interval). CVD, cardio-cerebrovascular disease; PxM, prescription-based methodology; PDC_with≥1_, proportion of days covered with at least one drug; PDC_wm_, duration weighted mean PDC; DPPR, daily polypharmacy possession ratio; FxM, fixed period-based methodology.

**Table 4.** Concordance indexes for the evaluation of time-to-event models of the predefined six methodologic measurements in this study

Variables			Unadjusted model			Fully adjusted model		
			AIC	Harrell’s C-statistics (95% CI)	Uno’s C-statistics (95% CI)	AIC	Harrell’s C-statistics (95% CI)	Uno’s C-statistics (95% CI)
All-cause mortality and CVD-specific hospitalization								
	PxM							
		PDC_with≥1_	12,902.18	0.528 (0.511, 0.546)	0.530 (0.459, 0.602)	12,384.36	0.724 (0.707, 0.742)	0.718 (0.655, 0.781)
		PDC_wm_	12,900.91	0.530 (0.513, 0.548)	0.532 (0.454, 0.610)	12,385.41	0.725 (0.707, 0.742)	0.718 (0.651, 0.784)
		DPPR	12,901.98	0.529 (0.512, 0.546)	0.531 (0.455, 0.606)	12,384.77	0.724 (0.707, 0.742)	0.718 (0.644, 0.791)
	FxM							
		PDC_with≥1_	12,899.94	0.533 (0.515, 0.550)	0.534 (0.453, 0.614)	12,379.55	0.725 (0.708, 0.743)	0.719 (0.648, 0.790)
		PDC_wm_	12,897.94	0.535 (0.517, 0.553)	0.536 (0.452, 0.619)	12,378.87	0.726 (0.708, 0.743)	0.719 (0.646, 0.792)
		DPPR	12,900.77	0.532 (0.514, 0.549)	0.533 (0.452, 0.613)	12,381.28	0.725 (0.707, 0.743)	0.719 (0.656, 0.781)
All-cause mortality								
	PxM							
		PDC_with≥1_	4,641.99	0.541 (0.511, 0.570)	0.537 (0.438, 0.637)	4,141.17	0.838 (0.813, 0.863)	0.842 (0.756, 0.929)
		PDC_wm_	4,639.54	0.549 (0.519, 0.578)	0.542 (0.442, 0.643)	4,137.94	0.839 (0.814, 0.865)	0.843 (0.743, 0.943)
		DPPR	4,641.50	0.542 (0.513, 0.571)	0.539 (0.434, 0.643)	4,140.74	0.838 (0.812, 0.863)	0.842 (0.743, 0.941)
	FxM							
		PDC_with≥1_	4,638.92	0.549 (0.520, 0.579)	0.548 (0.429, 0.667)	4,137.03	0.838 (0.812, 0.863)	0.842 (0.732, 0.951)
		PDC_wm_	4,637.27	0.554 (0.525, 0.583)	0.550 (0.430, 0.670)	4,134.34	0.839 (0.814, 0.865)	0.843 (0.735, 0.951)
		DPPR	4,639.61	0.548 (0.518, 0.577)	0.546 (0.429, 0.663)	4,137.77	0.838 (0.812, 0.863)	0.842 (0.713, 0.970)
CVD-specific mortality								
	PxM							
		PDC_with≥1_	1,123.75	0.571 (0.511, 0.631)	0.563 (0.377, 0.748)	1,026.53	0.845 (0.795, 0.895)	0.842 (0.700, 0.984)
		PDC_wm_	1,121.28	0.585 (0.526, 0.643)	0.582 (0.369, 0.795)	1,024.38	0.848 (0.799, 0.897)	0.846 (0.716, 0.977)
		DPPR	1,124.10	0.569 (0.509, 0.629)	0.560 (0.390, 0.731)	1,026.93	0.845 (0.795, 0.895)	0.841 (0.715, 0.968)
	FxM							
		PDC_with≥1_	1,119.62	0.597 (0.539, 0.656)	0.585 (0.368, 0.801)	1,021.00	0.849 (0.798, 0.899)	0.844 (0.700, 0.988)
		PDC_wm_	1,118.51	0.600 (0.544, 0.656)	0.594 (0.356, 0.832)	1,020.58	0.850 (0.801, 0.900)	0.848 (0.721, 0.975)
		DPPR	1,119.95	0.596 (0.537, 0.654)	0.583 (0.386, 0.781)	1,021.40	0.849 (0.798, 0.899)	0.844 (0.715, 0.972)
CVD-specific hospitalization								
	PxM							
		PDC_with≥1_	10,566.15	0.532 (0.513, 0.551)	0.535 (0.453, 0.617)	10,239.35	0.706 (0.686, 0.725)	0.698 (0.628, 0.767)
		PDC_wm_	10,566.18	0.532 (0.513, 0.551)	0.535 (0.453, 0.617)	10,242.13	0.705 (0.685, 0.725)	0.696 (0.622, 0.770)
		DPPR	10,566.63	0.531 (0.512, 0.550)	0.534 (0.449, 0.619)	10,240.71	0.706 (0.686, 0.725)	0.697 (0.625, 0.770)
	FxM							
		PDC_with≥1_	10,566.96	0.532 (0.512, 0.551)	0.535 (0.447, 0.622)	10,239.36	0.706 (0.687, 0.726)	0.699 (0.639, 0.758)
		PDC_wm_	10,566.00	0.533 (0.514, 0.552)	0.536 (0.451, 0.620)	10,239.87	0.706 (0.687, 0.726)	0.698 (0.621, 0.774)
		DPPR	10,567.55	0.531 (0.511, 0.550)	0.534 (0.451, 0.616)	10,240.71	0.706 (0.687, 0.726)	0.698 (0.617, 0.779)

AIC, Akaike information criterion; CI, confidence interval; CVD, cardio-cerebrovascular disease; PxM, prescription-based methodology; PDC_with≥1_, proportion of days covered with at least one drug; PDC_wm_, duration weighted mean PDC; DPPR, daily polypharmacy possession ratio; FxM, fixed period-based methodology.

## SUPPLEMENTARY MATERIALS

Supplementary Material 1. Patterns of antihypertensive multidrug therapy using a hypothetical patient case for measuring adherence estimates by the predefined six measurements

Supplementary Material 2 Baseline characteristics for adherent and non-adherent group by PxM-PDC_with≥1_

Supplementary Material 3 Baseline characteristics for adherent and non-adherent group by PxM-PDC_wm_

Supplementary Material 4 Baseline characteristics for adherent and non-adherent group by PxM-DPPR

Supplementary Material 5 Baseline characteristics for adherent and non-adherent group by FxM-PDC_with≥1_

Supplementary Material 6 Baseline characteristics for adherent and non-adherent group by FxM-PDC_wm_

Supplementary Material 7 Baseline characteristics for adherent and non-adherent group by FxM-DPPR

Supplementary Material 8 Estimates of adherence level and percentage of patients stratified by sex according to the predefined six methodologic measurements

Supplementary Material 9 Differences in Uno's Concordance Statistic for the primary outcome, a composite of all-cause mortality and CVD-specific hospitalization
