# Supplementary material for: A comparison of methods for the measurement of adherence to antihypertensive multidrug therapy and the clinical consequences: a retrospective cohort study using the Korean nationwide claims database
Source: Epidemiol Health. 2023 May 1;45:e2023050. doi: 10.4178/epih.e2023050 (PMC10593586; doi:10.4178/epih.e2023050)
Supplement: Supplementary Material 1. — Patterns of antihypertensive multidrug therapy using a hypothetical patient case for measuring adherence estimates by the predefined six measurements [file epih-45-e2023050-Supplementary-1.docx]

**Supplementary Material 1. Patterns of antihypertensive multidrug therapy using a hypothetical patient case for measuring adherence estimates by the predefined six measurements**

**
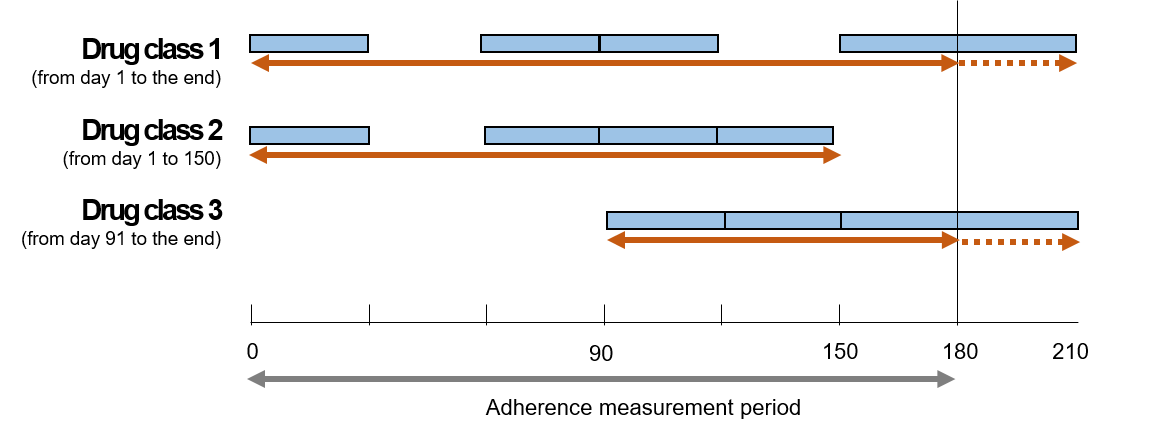
**

| **Prescription-based methodology (PxM)** | |
| --- | --- |
| **PDC_with≥1_** | = 180 days covered / 210 days = **85.7%** |
| **PDC_wm_** | = (71.43% $\times$ 210 + 80% $\times$ 150 + 100% $\times$ 120) / (210 + 150 + 60) = **81.25 %**   - PDC of class 1 = (150 days covered / 210 dyas) _day 1 to 210_ = 71.43% - PDC of class 2 = (120 days covered / 150 dyas) _day 1 to 150_ = 80% - PDC of class 3 = (120 days covered / 120 dyas) _day 91 to 210_ = 100% |
| **DPPR** | = [(2/$2\times3$0)_day 1 to 30_$+$(0/2$\times3$0) _day 31 to 60_$+$(2/2$\times3$0)$\text{day 61 to 90}+$  (3/$3\times3$0) _day 91 to 120_ $+$(2/3$\times3$0) _day 121 to 150_ $+$(2/2$\times6$0) _day 151 to 210_] / 210  = **80.95%** |
| **Fixed period-based methodology (FxM)** | |
| **PDC_with≥1_** | = 150 days covered / 180 days = **83.3%** |
| **PDC_wm_** | = (66.67% $\times$ 180 + 80% $\times$ 150 + 100% $\times$ 90) / (180 + 150 + 90) = **78.57 %**   - PDC of class 1 = (120 days covered / 180 dyas) _day 1 to 180_ = 66.67% - PDC of class 2 = (120 days covered / 150 dyas) _day 1 to 150_ = 80% - PDC of class 3 = (90 days covered / 90 dyas) _day 91 to 180_ = 100% |
| **DPPR** | = [(2/$2\times3$0)_day 1 to 30_$+$(0/2$\times3$0) _day 31 to 60_$+$(2/2$\times3$0)$\text{day 61 to 90}+$  (3/$3\times3$0) _day 91 to 120_ $+$(2/3$\times3$0) _day 121 to 150_ $+$(2/2$\times$30) _day 151 to 180_] / 180  = **77.78%** |

Abbreviation: DPPR, daily polypharmacy possession ratio; FxM, fixed period-based methodology; PDC_with≥1_, proportion of days covered with at least one drug; PDC_wm_, duration weighted mean proportion of days covered; PxM, prescription-based methodology
