# Supplementary material for: A comparison of methods for the measurement of adherence to antihypertensive multidrug therapy and the clinical consequences: a retrospective cohort study using the Korean nationwide claims database
Source: Epidemiol Health. 2023 May 1;45:e2023050. doi: 10.4178/epih.e2023050 (PMC10593586; doi:10.4178/epih.e2023050)
Supplement: Supplementary Material 2 — Baseline characteristics for adherent and non-adherent group by PxM-PDCwith≥1 [file epih-45-e2023050-Supplementary-2.docx]

**Supplementary Material 2. Baseline characteristics for adherent and non-adherent group by PxM-PDC_with≥1_**

| **Characteristic** | | **Adherent** | | **Non-adherent** | | **p-value** |
| --- | --- | --- | --- | --- | --- | --- |
|  | | **N** | **( % )** | **N** | **( % )** |  |
| Overall |  | 2,739 | (64.8) | 1,487 | (35.2) |  |
| Sex | Male | 1,432 | (52.3) | 792 | (53.3) | 0.54 |
|  | Female | 1,307 | (47.7) | 695 | (46.7) |  |
| Age | mean ± SD | 54.78 | ±13.51 | 56.23 | ±11.88 |  |
|  | 20-39 | 198 | (7.2) | 186 | (12.5) | <0.01 |
|  | 40-49 | 649 | (23.7) | 401 | (27.0) |  |
|  | 50-59 | 854 | (31.2) | 366 | (24.6) |  |
|  | 60-69 | 633 | (23.1) | 295 | (19.8) |  |
|  | 70+ | 405 | (14.8) | 239 | (16.1) |  |
| Disability |  | 205 | (7.5) | 109 | (7.3) | 0.86 |
| Type of health insurance | National Health Insurance | 2,585 | (94.4) | 1,403 | (94.4) | 0.97 |
|  | Medical aid | 154 | (5.6) | 84 | (5.6) |  |
| Socio-economic status | High | 1,076 | (39.3) | 525 | (35.3) | 0.05 |
|  | Middle | 892 | (32.6) | 539 | (36.2) |  |
|  | Low | 593 | (21.7) | 326 | (21.9) |  |
|  | Missing data | 178 | (6.5) | 97 | (6.5) |  |
| Medical institution type | Tertiary | 141 | (5.1) | 45 | (3.0) | <0.01 |
|  | Secondary | 295 | (10.8) | 137 | (9.2) |  |
|  | Clinic | 2,050 | (74.8) | 1,172 | (78.8) |  |
|  | Public health center | 253 | (9.2) | 133 | (8.9) |  |
| No. of AHTN classes | 2 | 2,102 | (76.7) | 1,171 | (78.7) | 0.14 |
|  | 3+ | 637 | (23.3) | 316 | (21.3) |  |
| Charlson Comorbidity Index | 0 | 2,016 | (73.6) | 1,055 | (70.9) | 0.10 |
|  | 1 | 509 | (18.6) | 291 | (19.6) |  |
|  | 2+ | 214 | (7.8) | 141 | (9.5) |  |
| Diabetes |  | 461 | (16.8) | 191 | (12.8) | <0.01 |
| Dyslipidemia |  | 923 | (33.7) | 385 | (25.9) | <0.01 |

Abbreviation: AHTN, antihypertensive agents; PxM, prescription-based methodology; PDC_with≥1_, proportion of days covered with at least one drug.
