# Supplementary material for: A comparison of methods for the measurement of adherence to antihypertensive multidrug therapy and the clinical consequences: a retrospective cohort study using the Korean nationwide claims database
Source: Epidemiol Health. 2023 May 1;45:e2023050. doi: 10.4178/epih.e2023050 (PMC10593586; doi:10.4178/epih.e2023050)
Supplement: Supplementary Material 3 — Baseline characteristics for adherent and non-adherent group by PxM-PDCwm [file epih-45-e2023050-Supplementary-3.docx]

**Supplementary Material 3. Baseline characteristics for adherent and non-adherent group by PxM-PDC_wm_**

| **Characteristic** | | **Adherent** | | **Non-adherent** | | **p-value** |
| --- | --- | --- | --- | --- | --- | --- |
|  | | **N** | **( % )** | **N** | **( % )** |  |
| Overall |  | 2,440 | (57.7) | 1,786 | (42.3) |  |
| Sex | Male | 1,269 | (52.0) | 955 | (53.5) | 0.35 |
|  | Female | 1,171 | (48.0) | 831 | (46.5) |  |
| Age | mean ± SD | 55.08 | ±13.21 | 56.20 | ±11.92 |  |
|  | 20-39 | 185 | (7.6) | 199 | (11.1) | <0.01 |
|  | 40-49 | 569 | (23.3) | 481 | (26.9) |  |
|  | 50-59 | 764 | (31.3) | 456 | (25.5) |  |
|  | 60-69 | 570 | (23.4) | 358 | (20.0) |  |
|  | 70+ | 352 | (14.4) | 292 | (16.3) |  |
| Disability |  | 175 | (7.2) | 139 | (7.8) | 0.45 |
| Type of health insurance | National Health Insurance | 2,307 | (94.5) | 1,681 | (94.1) | 0.55 |
|  | Medical aid | 133 | (5.5) | 105 | (5.9) |  |
| Socio-economic status | High | 954 | (39.1) | 647 | (36.2) | 0.07 |
|  | Middle | 789 | (32.3) | 642 | (35.9) |  |
|  | Low | 542 | (22.2) | 377 | (21.1) |  |
|  | Missing data | 155 | (6.4) | 120 | (6.7) |  |
| Medical institution type | Tertiary | 128 | (5.2) | 58 | (3.2) | <0.01 |
|  | Secondary | 273 | (11.2) | 159 | (8.9) |  |
|  | Clinic | 1,827 | (74.9) | 1,395 | (78.1) |  |
|  | Public health center | 212 | (8.7) | 174 | (9.7) |  |
| No. of AHTN classes | 2 | 1,881 | (77.1) | 1,392 | (77.9) | 0.51 |
|  | 3+ | 559 | (22.9) | 394 | (22.1) |  |
| Charlson Comorbidity Index | 0 | 1,809 | (74.1) | 1,262 | (70.7) | 0.04 |
|  | 1 | 441 | (18.1) | 359 | (20.1) |  |
|  | 2+ | 190 | (7.8) | 165 | (9.2) |  |
| Diabetes |  | 404 | (16.6) | 248 | (13.9) | 0.02 |
| Dyslipidemia |  | 826 | (33.9) | 482 | (27.0) | <0.01 |

Abbreviation: AHTN, antihypertensive agents; PxM, prescription-based methodology; PDC_wm_, duration weighted mean PDC.
