# Supplementary material for: A comparison of methods for the measurement of adherence to antihypertensive multidrug therapy and the clinical consequences: a retrospective cohort study using the Korean nationwide claims database
Source: Epidemiol Health. 2023 May 1;45:e2023050. doi: 10.4178/epih.e2023050 (PMC10593586; doi:10.4178/epih.e2023050)
Supplement: Supplementary Material 4 — Baseline characteristics for adherent and non-adherent group by PxM-DPPR [file epih-45-e2023050-Supplementary-4.docx]

**Supplementary Material 4. Baseline characteristics for adherent and non-adherent group by PxM-DPPR**

| **Characteristic** | | **Adherent** | | **Non-adherent** | | **p-value** |
| --- | --- | --- | --- | --- | --- | --- |
|  | | **N** | **( % )** | **N** | **( % )** |  |
| Overall |  | 2,720 | (64.4) | 1,506 | (35.6) |  |
| Sex | Male | 1,422 | (52.3) | 802 | (53.3) | 0.54 |
|  | Female | 1,298 | (47.7) | 704 | (46.7) |  |
| Age | mean ± SD | 54.80 | ±13.53 | 56.23 | ±11.85 |  |
|  | 20-39 | 196 | (7.2) | 188 | (12.5) | <0.01 |
|  | 40-49 | 644 | (23.7) | 406 | (27.0) |  |
|  | 50-59 | 850 | (31.3) | 370 | (24.6) |  |
|  | 60-69 | 629 | (23.1) | 299 | (19.9) |  |
|  | 70+ | 401 | (14.7) | 243 | (16.1) |  |
| Disability |  | 199 | (7.3) | 115 | (7.6) | 0.70 |
| Type of health insurance | National Health Insurance | 2,567 | (94.4) | 1,421 | (94.4) | 0.98 |
|  | Medical aid | 153 | (5.6) | 85 | (5.6) |  |
| Socio-economic status | High | 1,069 | (39.3) | 532 | (35.3) | 0.04 |
|  | Middle | 883 | (32.5) | 548 | (36.4) |  |
|  | Low | 591 | (21.7) | 328 | (21.8) |  |
|  | Missing data | 177 | (6.5) | 98 | (6.5) |  |
| Medical institution type | Tertiary | 140 | (5.1) | 46 | (3.1) | 0.00 |
|  | Secondary | 295 | (10.8) | 137 | (9.1) |  |
|  | Clinic | 2,032 | (74.7) | 1,190 | (79.0) |  |
|  | Public health center | 253 | (9.3) | 133 | (8.8) |  |
| No. of AHTN classes | 2 | 2,088 | (76.8) | 1,185 | (78.7) | 0.15 |
|  | 3+ | 632 | (23.2) | 321 | (21.3) |  |
| Charlson Comorbidity Index | 0 | 2,005 | (73.7) | 1,066 | (70.8) | 0.06 |
|  | 1 | 504 | (18.5) | 296 | (19.7) |  |
|  | 2+ | 211 | (7.8) | 144 | (9.6) |  |
| Diabetes |  | 455 | (16.7) | 197 | (13.1) | <0.01 |
| Dyslipidemia |  | 915 | (33.6) | 393 | (26.1) | <0.01 |

Abbreviation: AHTN, antihypertensive agents; PxM, prescription-based methodology; DPPR, daily polypharmacy possession ratio.
