# Supplementary material for: A comparison of methods for the measurement of adherence to antihypertensive multidrug therapy and the clinical consequences: a retrospective cohort study using the Korean nationwide claims database
Source: Epidemiol Health. 2023 May 1;45:e2023050. doi: 10.4178/epih.e2023050 (PMC10593586; doi:10.4178/epih.e2023050)
Supplement: Supplementary Material 5 — Baseline characteristics for adherent and non-adherent group by FxM-PDCwith≥1 [file epih-45-e2023050-Supplementary-5.docx]

**Supplementary Material 5. Baseline characteristics for adherent and non-adherent group by FxM-PDC_with≥1_**

| **Characteristic** | | **Adherent** | | **Non-adherent** | | **p-value** |
| --- | --- | --- | --- | --- | --- | --- |
|  | | **N** | **( % )** | **N** | **( % )** |  |
| Overall |  | 2,506 | (59.3) | 1,720 | (40.7) |  |
| Sex | Male | 1,299 | (51.8) | 925 | (53.8) | 0.21 |
|  | Female | 1,207 | (48.2) | 795 | (46.2) |  |
| Age | mean ± SD | 54.71 | ±13.62 | 56.42 | ±11.6 |  |
|  | 20-39 | 165 | (6.6) | 219 | (12.7) | <0.01 |
|  | 40-49 | 591 | (23.6) | 459 | (26.7) |  |
|  | 50-59 | 793 | (31.6) | 427 | (24.8) |  |
|  | 60-69 | 589 | (23.5) | 339 | (19.7) |  |
|  | 70+ | 368 | (14.7) | 276 | (16.0) |  |
| Disability |  | 188 | (7.5) | 126 | (7.3) | 0.83 |
| Type of health insurance | National Health Insurance | 2,361 | (94.2) | 1,627 | (94.6) | 0.60 |
|  | Medical aid | 145 | (5.8) | 93 | (5.4) |  |
| Socio-economic status | High | 983 | (39.2) | 618 | (35.9) | 0.07 |
|  | Middle | 814 | (32.5) | 617 | (35.9) |  |
|  | Low | 540 | (21.5) | 379 | (22.0) |  |
|  | Missing data | 169 | (6.7) | 106 | (6.2) |  |
| Medical institution type | Tertiary | 123 | (4.9) | 63 | (3.7) | 0.26 |
|  | Secondary | 259 | (10.3) | 173 | (10.1) |  |
|  | Clinic | 1,895 | (75.6) | 1,327 | (77.2) |  |
|  | Public health center | 229 | (9.1) | 157 | (9.1) |  |
| No. of AHTN classes | 2 | 1,912 | (76.3) | 1,361 | (79.1) | 0.03 |
|  | 3+ | 594 | (23.7) | 359 | (20.9) |  |
| Charlson Comorbidity Index | 0 | 1,854 | (74.0) | 1,217 | (70.8) | 0.07 |
|  | 1 | 450 | (18.0) | 350 | (20.3) |  |
|  | 2+ | 202 | (8.1) | 153 | (8.9) |  |
| Diabetes |  | 426 | (17.0) | 226 | (13.1) | <0.01 |
| Dyslipidemia |  | 853 | (34.0) | 455 | (26.5) | <0.01 |

Abbreviation: AHTN, antihypertensive agents; FxM, fixed period-based methodology; PDC_with≥1_, proportion of days covered with at least one drug.
