# Supplementary material for: A comparison of methods for the measurement of adherence to antihypertensive multidrug therapy and the clinical consequences: a retrospective cohort study using the Korean nationwide claims database
Source: Epidemiol Health. 2023 May 1;45:e2023050. doi: 10.4178/epih.e2023050 (PMC10593586; doi:10.4178/epih.e2023050)
Supplement: Supplementary Material 6 — Baseline characteristics for adherent and non-adherent group by FxM-PDCwm [file epih-45-e2023050-Supplementary-6.docx]

**Supplementary Material 6. Baseline characteristics for adherent and non-adherent group by FxM-PDC_wm_**

| **Characteristic** | | **Adherent** | | **Non-adherent** | | **p-value** |
| --- | --- | --- | --- | --- | --- | --- |
|  | | **N** | **( % )** | **N** | **( % )** |  |
| Overall |  | 2,240 | (53.0) | 1,986 | (47.0) |  |
| Sex | Male | 1,155 | (51.6) | 1,069 | (53.8) | 0.14 |
|  | Female | 1,085 | (48.4) | 917 | (46.2) |  |
| Age | mean ± SD | 54.91 | ±13.35 | 56.44 | ±11.63 |  |
|  | 20-39 | 152 | (6.8) | 232 | (11.7) | <0.01 |
|  | 40-49 | 519 | (23.2) | 531 | (26.7) |  |
|  | 50-59 | 710 | (31.7) | 510 | (25.7) |  |
|  | 60-69 | 537 | (24.0) | 391 | (19.7) |  |
|  | 70+ | 322 | (14.4) | 322 | (16.2) |  |
| Disability |  | 164 | (7.3) | 150 | (7.6) | 0.77 |
| Type of health insurance | National Health Insurance | 2,114 | (94.4) | 1,874 | (94.4) | 0.98 |
|  | Medical aid | 126 | (5.6) | 112 | (5.6) |  |
| Socio-economic status | High | 883 | (39.4) | 718 | (36.2) | 0.07 |
|  | Middle | 721 | (32.2) | 710 | (35.8) |  |
|  | Low | 488 | (21.8) | 431 | (21.7) |  |
|  | Missing data | 148 | (6.6) | 127 | (6.4) |  |
| Medical institution type | Tertiary | 113 | (5.0) | 73 | (3.7) | 0.04 |
|  | Secondary | 243 | (10.8) | 189 | (9.5) |  |
|  | Clinic | 1,693 | (75.6) | 1,529 | (77.0) |  |
|  | Public health center | 191 | (8.5) | 195 | (9.8) |  |
| No. of AHTN classes | 2 | 1,713 | (76.5) | 1,560 | (78.5) | 0.11 |
|  | 3+ | 527 | (23.5) | 426 | (21.5) |  |
| Charlson Comorbidity Index | 0 | 1,669 | (74.5) | 1,402 | (70.6) | 0.02 |
|  | 1 | 393 | (17.5) | 407 | (20.5) |  |
|  | 2+ | 178 | (7.9) | 177 | (8.9) |  |
| Diabetes |  | 369 | (16.5) | 283 | (14.2) | 0.05 |
| Dyslipidemia |  | 765 | (34.2) | 543 | (27.3) | <0.01 |

Abbreviation: AHTN, antihypertensive agents; FxM, fixed period-based methodology; PDC_wm_, duration weighted mean PDC.
