# Supplementary material for: A comparison of methods for the measurement of adherence to antihypertensive multidrug therapy and the clinical consequences: a retrospective cohort study using the Korean nationwide claims database
Source: Epidemiol Health. 2023 May 1;45:e2023050. doi: 10.4178/epih.e2023050 (PMC10593586; doi:10.4178/epih.e2023050)
Supplement: Supplementary Material 7 — Baseline characteristics for adherent and non-adherent group by FxM-DPPR [file epih-45-e2023050-Supplementary-7.docx]

**Supplementary Material 7. Baseline characteristics for adherent and non-adherent group by FxM-DPPR**

| **Characteristic** | | **Adherent** | | **Non-adherent** | | **p-value** |
| --- | --- | --- | --- | --- | --- | --- |
|  | | **N** | **( % )** | **N** | **( % )** |  |
| Overall |  | 2,493 | (59.0) | 1,733 | (41.0) |  |
| Sex | Male | 1,291 | (51.8) | 933 | (53.8) | 0.19 |
|  | Female | 1,202 | (48.2) | 800 | (46.2) |  |
| Age | mean ± SD | 54.70 | ±13.63 | 56.44 | ±11.59 |  |
|  | 20-39 | 163 | (6.5) | 221 | (12.8) | <0.01 |
|  | 40-49 | 588 | (23.6) | 462 | (26.7) |  |
|  | 50-59 | 790 | (31.7) | 430 | (24.8) |  |
|  | 60-69 | 587 | (23.5) | 341 | (19.7) |  |
|  | 70+ | 365 | (14.6) | 279 | (16.1) |  |
| Disability |  | 185 | (7.4) | 129 | (7.4) | 0.98 |
| Type of health insurance | National Health Insurance | 2,349 | (94.2) | 1,639 | (94.6) | 0.63 |
|  | Medical aid | 144 | (5.8) | 94 | (5.4) |  |
| Socio-economic status | High | 981 | (39.4) | 620 | (35.8) | 0.04 |
|  | Middle | 807 | (32.4) | 624 | (36.0) |  |
|  | Low | 537 | (21.5) | 382 | (22.0) |  |
|  | Missing data | 168 | (6.7) | 107 | (6.2) |  |
| Medical institution type | Tertiary | 123 | (4.9) | 63 | (3.6) | 0.21 |
|  | Secondary | 259 | (10.4) | 173 | (10.0) |  |
|  | Clinic | 1,882 | (75.5) | 1,340 | (77.3) |  |
|  | Public health center | 229 | (9.2) | 157 | (9.1) |  |
| No. of AHTN classes | 2 | 1,903 | (76.3) | 1,370 | (79.1) | 0.04 |
|  | 3+ | 590 | (23.7) | 363 | (20.9) |  |
| Charlson Comorbidity Index | 0 | 1,845 | (74.0) | 1,226 | (70.7) | 0.06 |
|  | 1 | 447 | (17.9) | 353 | (20.4) |  |
|  | 2+ | 201 | (8.1) | 154 | (8.9) |  |
| Diabetes |  | 421 | (16.9) | 231 | (13.3) | <0.01 |
| Dyslipidemia |  | 846 | (33.9) | 462 | (26.7) | <0.01 |

Abbreviation: AHTN, antihypertensive agents; FxM, fixed period-based methodology; DPPR, daily polypharmacy possession ratio.
