# Supplementary material for: A comparison of methods for the measurement of adherence to antihypertensive multidrug therapy and the clinical consequences: a retrospective cohort study using the Korean nationwide claims database
Source: Epidemiol Health. 2023 May 1;45:e2023050. doi: 10.4178/epih.e2023050 (PMC10593586; doi:10.4178/epih.e2023050)
Supplement: Supplementary Material 8 — Estimates of adherence level and percentage of patients stratified by sex according to the predefined six methodologic measurements [file epih-45-e2023050-Supplementary-8.docx]

**Supplementary Material 8. Estimates of adherence level and percentage of patients stratified by sex according to the predefined six methodologic measurements**


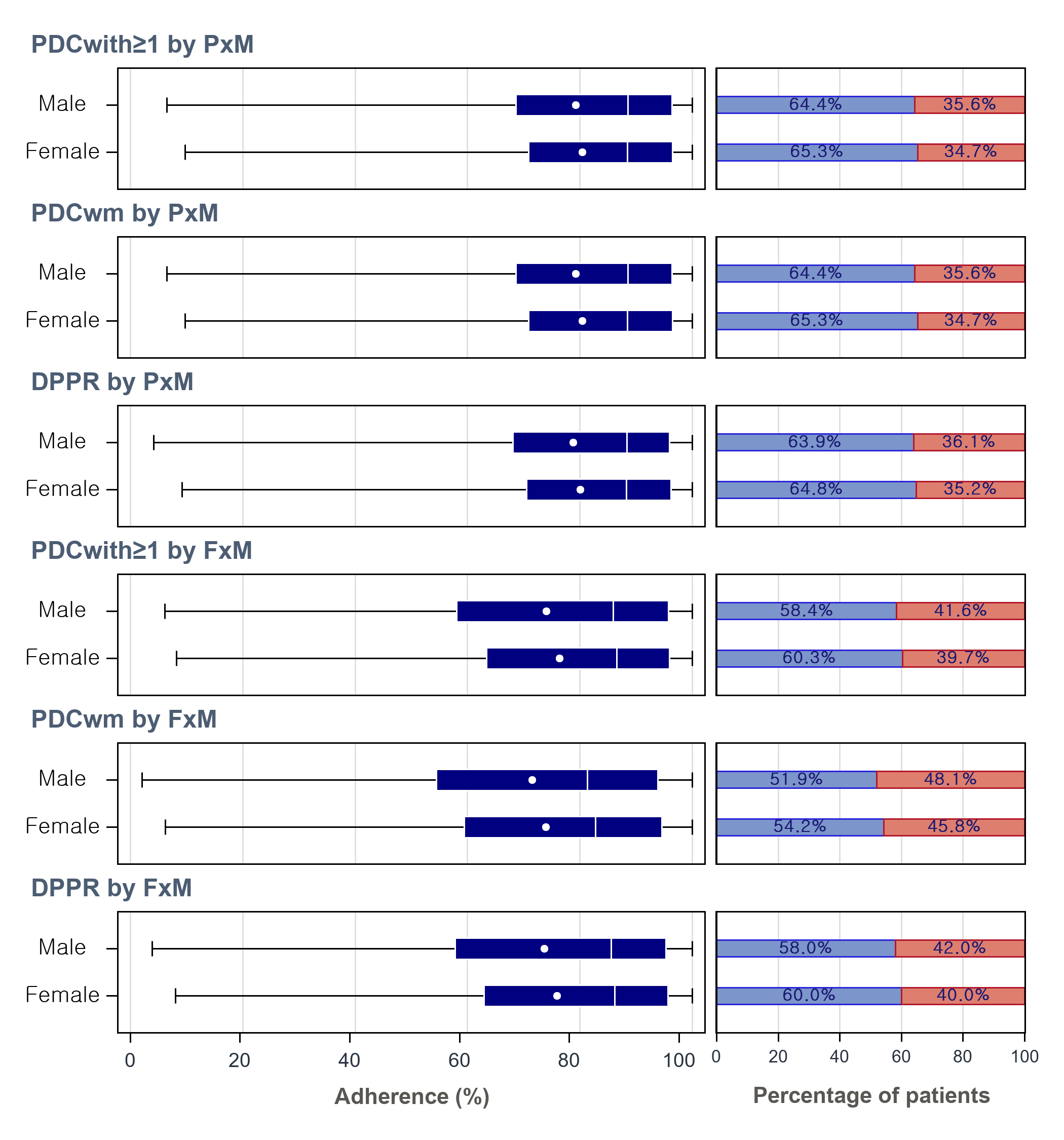


Abbreviation: DPPR, daily polypharmacy possession ratio; FxM, fixed period-based methodology; PDC_with≥1_, proportion of days covered with at least one drug; PDC_wm_, duration weighted mean PDC; PxM, prescription-based methodology.
