# Supplementary material for: A comparison of methods for the measurement of adherence to antihypertensive multidrug therapy and the clinical consequences: a retrospective cohort study using the Korean nationwide claims database
Source: Epidemiol Health. 2023 May 1;45:e2023050. doi: 10.4178/epih.e2023050 (PMC10593586; doi:10.4178/epih.e2023050)
Supplement: Supplementary Material 9 — Differences in Uno's Concordance Statistic for the primary outcome, a composite of all-cause mortality and CVD-specific hospitalization [file epih-45-e2023050-Supplementary-9.docx]

**Supplementary Material 9. Differences in Uno's Concordance Statistic for the primary outcome, a composite of all-cause mortality and CVD-specific hospitalization**

|  |  | Base  Model^a^ | PxM | | | FxM | | |
| --- | --- | --- | --- | --- | --- | --- | --- | --- |
|  |  |  | PDC_with≥1_ | PDC_wm_ | DPPR | PDC_with≥1_ | PDC_wm_ | DPPR |
| PxM | PDC_with≥1_ | <0.0001 | . | 1 | 1 | 0.2691 | 0.2691 | 0.2691 |
|  | PDC_wm_ | <0.0001 |  | . | 1 | 0.2691 | 0.2691 | 0.2691 |
|  | DPPR | <0.0001 |  |  | . | 0.2691 | 0.2691 | 0.2691 |
| FxM | PDC_with≥1_ | <0.0001 |  |  |  | . | 1 | 1 |
|  | PDC_wm_ | <0.0001 |  |  |  |  | . | 1 |
|  | DPPR | <0.0001 |  |  |  |  |  | . |
| ^a^ To evaluate effects of different measurements of adherence, the base model included sex, age, disability, type of health insurance, socioeconomic status, type of medical institution, type of multidrug therapy, Charlson comorbidity index, and a history of diabetes mellitus and dyslipidemia, except for the dichotomous variables of adherence such as PDC_with≥1,_ PDC_wm,_ and DPPR. | | | | | | | | |

Abbreviation: CVD, cardiovascular disease; DPPR, daily polypharmacy possession ratio; FxM, fixed period-based methodology; PDC_with≥1_, proportion of days covered with at least one drug; PDC_wm_, duration weighted mean PDC; PxM, prescription-based methodology.
